# Supplementary material for: Magnitude of metabolic syndrome in Gondar town, Northwest Ethiopia: A community-based cross-sectional study
Source: PLoS One. 2021 Oct 7;16(10):e0257306. doi: 10.1371/journal.pone.0257306 (PMC8496848; doi:10.1371/journal.pone.0257306)
Supplement: S1 Table — (DOCX) [file pone.0257306.s001.docx]

**S1 Table. Characteristics of CVD Risk factors by sex and age among Gondar city residents who were ≥18 years old, Northwest Ethiopia**

| **Variable** | **Sex** | **Age in Year** | | | | | |  |
| --- | --- | --- | --- | --- | --- | --- | --- | --- |
|  |  | **18-24** | **25-34** | **35-44** | **45-54** | **55-64** | **65&above** | **Total** |
| Under weight | Male | 57 (21.59) | 34 (11.15) | 29 (11.46) | 24 (10.17) | 23 (14.11) | 15 (8.11) | 182 (12.94) |
|  | Female | 56 (18.98) | 47 (13.54) | 43 (13.03) | 25 (9.12) | 26 (11.66) | 27 (14.67) | 224 (13.55) |
| Normal | Male | 176 (66.67) | 209 (68.52) | 164 (64.82) | 157 (66.53) | 104 (63.8) | 122 (65.95) | 932 (66.29) |
|  | Female | 189 (64.07) | 213 (61.38) | 176 (53.33) | 163 (59.49) | 122 (54.71) | 101 (54.89) | 964 (58.32) |
| Over weight | Male | 24 (9.09) | 52 (17.05) | 54 (21.34) | 45 (19.07) | 31 (19.02) | 44 (23.78) | 250 (17.78) |
|  | Female | 43 (14.58) | 66 (19.02) | 75 (22.73) | 62 (22.63) | 52 (23.32) | 35 (19.02) | 333 (20.15) |
| Obese | Male | 7 (2.65) | 10 (3.28) | 6 (2.37) | 10 (4.24) | 5 (3.07) | 4 (2.16) | 42 (2.99) |
|  | Female | 7 (2.37) | 21 (6.05) | 36 (10.91) | 24 (8.76) | 23 (10.31) | 21 (11.41) | 132 (7.99) |
| Alcohol consumption in the last 30 days | Male | 145 (54.92) | 118 (38.69) | 85 (33.6) | 86 (36.44) | 70 (42.94) | 104 (56.22) | 608 (43.24) |
|  | Female | 81(27.46) | 142 (40.92) | 160 (48.48) | 126 (45.99) | 95 (42.6) | 68 (36.96) | 672 (40.65) |
| Current Smoking | Male | 5 (1.91) | 14 (4.62) | 9 (3.56) | 12 (5.08) | 5 (3.14) | 3 (1.62) | 48 (3.43) |
|  | Female | 1 (0.34) | 3 (0.87) | 1 (0.3) | 3 (1.12) | 0 (0.00) | 0 (0.00) | 8 (0.49) |
| Moderate exercise | Male | 65 (24.62) | 77 (25.25) | 65 (25.69) | 60 (25.42) | 21 (12.88) | 29 (15.68) | 317 (22.55) |
|  | Female | 77 (26.1) | 127 (36.6) | 102 (30.91) | 65 (23.72) | 55 (24.66) | 35 (19.02) | 461 (27.89) |
| Waist circumference | Male | 10 (3.79) | 25 (8.2) | 35 (13.83) | 39 (16.53) | 23 (14.11) | 32 (17.3) | 164 (11.66) |
|  | Female | 74 (25.08) | 104 (29.97) | 138 (41.82) | 126 (45.99) | 115 (51.57) | 102 (55.43) | 659 (39.87) |
