# Supplementary material for: Magnitude of metabolic syndrome in Gondar town, Northwest Ethiopia: A community-based cross-sectional study
Source: PLoS One. 2021 Oct 7;16(10):e0257306. doi: 10.1371/journal.pone.0257306 (PMC8496848; doi:10.1371/journal.pone.0257306)
Supplement: S1 File — (DOC) [file pone.0257306.s002.doc]

## Participant Identification Number ______

Guide to the columns

| Column | Description | Site Tailoring |
| --- | --- | --- |
| Number | This question reference number is designed to help interviewers find their place if interrupted. | Renumber the instrument sequentially once the content has been finalized. |
| Question | Each question is to be read to the participants | - Select sections to use. - Add expanded and optional questions as desired. |
| Response | This column lists the available response options which the interviewer will be circling or filling in the text boxes. The skip instructions are shown on the right hand side of the responses and should be carefully followed during interviews. | - Add site specific responses for demographic responses (e.g. C6). - Change skips question identifiers from code to question number. |
| Code | The column is designed to match data from the instrument into the data entry tool, data analysis syntax, data book, and fact sheet. | This should never be changed or removed. The code is used as a general identifier for the data entry and analysis. |

**PREVALENCE ASSESSMENT QUESTIONER**

WHO STEPS Instrument

Gondar, N.W. Ethiopia

| **Survey Information Section one to seven** | | | |
| --- | --- | --- | --- |
| Location and Date | | Response | Code |
| 1 | Cluster /Village ID | └─┴─┴─┘ | D1 |
| 2 | Cluster/kebele name |  | D2 |
| 3 | House ID | **└─┴─┴─┘** | D3 |
| 4 | Interviewer ID | └─┴─┴─┘ | D4 |
| 5 | Date of completion of the instrument | └─┴─┘ └─┴─┘ └─┴─┴─┴─┘ dd mm year | D5 |

*✁*

*✁*

| Consent, Interview Language and Name | | Response | | Code |
| --- | --- | --- | --- | --- |
| 6 | Interview Language *[Insert Language]* | English | 1 | D6 |
|  |  | *[Amharic]* | 2 |  |
|  |  | *Oromepha* | 3 |  |
|  |  | *Other* | 4 |  |
| **Additional Information that may be helpful** | | | | |
| 7 | Location of birth? : |  | | D7 |
| 8 | Religion | Orthodox 1  Muslim 2  Others 3 | | D8 |
| 9 | Time of interview  (24 hour clock) | └─┴─┘: └─┴─┘  hrs mins | | D9 |

| **CORE: Demographic Information** | | | | | |
| --- | --- | --- | --- | --- | --- |
| **Question** | | **Response** | | | **Code** |
| 10 | Sex (*Record Male / Female as observed)* | Male | 1 | | D10 |
|  |  | Female | 2 | |  |
| 11 | How old are you? | Years | | └─┴─┘ | D11 |
| 12 | What is the **highest level of education** you have completed?*[INSERT COUNTRY-SPECIFIC CATEGORIES]* | No formal schooling  Refused | | 1  88 | D12 |
| 13 | What is your *[insert relevant ethnic group / racial group / cultural subgroup / others]* **background**? | [*Amhara]*  [*Tigria]*  [*Oromo]*  Others  Refused | | 1  2  3  4  88 | D13 |
|  |  | Other Specify | |  |  |
| 14 | What is your current **marital status**? | Never married  Currently married  Separated  Divorced  Widowed  Refused | | 1  2  3  4  5  88 | D14 |
| 15 | Which of the following best describes your **main** **work** status over the past 12 months?  *[INSERT COUNTRY-SPECIFIC CATEGORIES]*  (*USE SHOWCARD)* | Government employee  Non-government employee  Self-employed  Non-paid  Student  Homemaker  Retired  Unemployed (able to work) Unemployed (unable to work (Refused) | | 1  2  3  4  5  6  7  8  9  88 | D15 |

| **Step 1 Behavioral Measurements** | | | | |
| --- | --- | --- | --- | --- |
| **CORE: Tobacco Use** | | | | |
| Now I am going to ask you some questions about various health behaviours. This includes things like smoking, drinking alcohol, eating fruits and vegetables and physical activity. Let's start with tobacco. | | | | |
| **Question** | | **Response** | | **Code** |
| 16 | Do you currently smoke any **tobacco products**, such as cigarettes, cigars or pipes?  *(USE SHOWCARD)* | Yes | 1 | T1 |
|  |  | No | 2 *I* |  |
| 17 | Do you currently smoke tobacco products **daily**? | Yes | 1 | T2 |
|  |  | No | 2 |  |
| 18 | How old were you when you **first started** smoking daily? | Age (years) | **└─┴─┘** | T3 |
|  |  | Don’t know 77 |  |  |

| **EXPANDED: Tobacco Use** | | | | | | |
| --- | --- | --- | --- | --- | --- | --- |
| **Question** | | | **Response** | | | **Code** |
| 19 | How old were you when you **stopped** smoking **daily**? | | Age (years) | | **└─┴─┘** | T4 |
|  |  |  | Don’t Know 77 | |  |  |
| 20 | Do you **currently use** any **smokeless tobacco** such as *[snuff,]*? *(USE SHOWCARD)* | | Yes | 1 | | T5 |
|  |  |  | No | 2 | |  |
| 21 | Do you **currently use** any **smokeless tobacco** such as *[chewing tobacco, betel]*? *(USE SHOWCARD* | | Yes | 1 | | T6 |
|  |  |  | No | 2 | |  |
| 22 | On average, how many cigarrettes **/ day** do you use | |  |  | | T7 |
| 3 | |  | | | | |
| 23 | During the past 7 days, on how many days did someone **in your home** smoke when you were present? | | Number of days | | **└─┴─┘** | T8 |
|  |  |  | Don't know 77 | |  |  |
| 24 | During the past 7 days, on how many days did someone smoke in closed areas **in your workplace** (in the building, in a work area or a specific office) when you were present? | | Number of days | | **└─┴─┘** | T9 |
|  |  |  | Don't know or don't  work in a closed area 77 | |  |  |

| **CORE: Alcohol Consumption** | | | | |
| --- | --- | --- | --- | --- |
| The next questions ask about the consumption of alcohol. | | | | |
| **Question** | | **Response** | | **Code** |
| 25 | Have you **ever** consumed an alcoholic drink such as beer, wine,tela, tej, local Areki, fermented cider or *[add other local examples]*?  *(USE SHOWCARD OR SHOW EXAMPLES)* | Yes | 1 | A1 |
|  |  | No | 2 |  |
| 26 | Have you consumed an alcoholic drink within the **past 12 months**? | Yes | 1 | A2 |
|  |  | No | 2 |  |
| 27 | During the past 12 months, **how frequently** have you had at least one alcoholic drink?  *(READ RESPONSES, USE SHOWCARD)* | Daily | 1 | A3 |
|  |  | 5-6 days per week | 2 |  |
|  |  | 1-4 days per week | 3 |  |
|  |  | 1-3 days per month | 4 |  |
|  |  | Less than once a month | 5 |  |
| 28 | Have you consumed an alcoholic drink within the **past 30 days**? | Yes | 1 | A4 |
|  |  | No | 2 |  |
| 29 | During the past 30 days, on how many **occasions** did you have at least one alcoholic drink? | Number  Don't know 77 | └─┴─┘ | A5 |
| 30 | During the past 30 days, when you drank alcohol, **on average**, how many **standard** **alcoholic** **drinks** did you have during one drinking occasion?  *(USE SHOWCARD)* | Number  Don't know 77 | └─┴─┘ | A6 |

| **EXPANDED: Alcohol Consumption** | | | | |
| --- | --- | --- | --- | --- |
| 31 | During the past 30 days, when you consumed an alcoholic drink, how often was it with meals? Please do not count snacks. | Usually with meals | 1 | A7 |
|  |  | Sometimes with meals | 2 |  |
|  |  | Rarely with meals | 3 |  |
|  |  | Never with meals | 4 |  |
| 32 | During each of the **past 7 days**, how many standard alcoholic drinks did you have each day?  *(USE SHOWCARD)*  *Don't Know 77* | Monday | └─┴─┘ | A8a |
|  |  | Tuesday | └─┴─┘ | A8b |
|  |  | Wednesday | └─┴─┘ | A8c |
|  |  | Thursday | └─┴─┘ | A8d |
|  |  | Friday | └─┴─┘ | A8e |
|  |  | Saturday | └─┴─┘ | A8f |
|  |  | Sunday | └─┴─┘ | A8g |

| **CORE: Diet** | | | | | | | | |
| --- | --- | --- | --- | --- | --- | --- | --- | --- |
| The next questions ask about the fruits and vegetables that you usually eat. I have a nutrition card here that shows you some examples of local fruits and vegetables. Each picture represents the size of a serving. As you answer these questions please think of a typical week in the last year. | | | | | | | | |
| **Question** | | | | **Response** | | | **Code** | |
| 33 | | In a typical week, on how many days do you **eat fruit**?  *(USE SHOWCARD)* | | Number of days Don't Know 77 | └─┴─┘ | | N1 | |
| 34 | | In a typical week, on how many days do you **eat vegetables**? *(USE SHOWCARD)* | | Number of days Don't Know 77 | └─┴─┘ | | N2 | |
| **EXPANDED: Diet** | | | | | | | |  |
| 35 | What type of **oil or fat is most often** used for meal preparation in your household?  *(USE SHOWCARD)*  *(SELECT ONLY ONE)* | | Vegetable oil | | | 1 | N3 | |
|  |  |  | Seed oil | | | 2 |  |  |
|  |  |  | Butter or ghee | | | 3 |  |  |
|  |  |  | Margarine | | | 4 |  |  |
|  |  |  | If Other please specify | | | 5  *_____________________* |  |  |
|  |  |  | None in particular | | | 6 |  |  |
|  |  |  | None used | | | 7 |  |  |
|  |  |  | Don’t know | | | 77 |  |  |
|  |  |  | Other | | | └─┴─┴─┴─┴─┴─┴ | N4 | |
| 36 | During the past 30 days, on how many **occasions** did you have take meat? | | Never 1  Daily 2  Weekly 3  Monthly 4 | | |  | N5 | |
| 37 | Do you fast | | Yes 1  No 2 | | |  | N6 | |
| 38 | If Yes, for haw long per /day | | Hours : minutes | | | └─┴─┘: └─┴─┘  hrs mins | N7 | |
| 39 | Haw many day/ Per Year | | Number of days | | | └─┴─┘ | N8 | |
| 40 | What type of food you eat during fasting period | | 1. Animal product(meat, milk fish) 2. Without animal product (vegetable cereals) 3. Both | | |  | N9 | |
| 41 | Do you eat enjera | | Yes 1  No 2 | | |  | N10 | |
| 42 | If Yes how many times a day? | | Number | | | └─┴─┘: | N11 | |
| 43 | What type of grains/cereal you use in making enjera | | 1. Teff 2. Dagusa 3. Rice 4. Mixed   If other pleas specify | | |  | N12 | |

| **CORE: Physical Activity** | | | | | |
| --- | --- | --- | --- | --- | --- |
| Next I am going to ask you about the time you spend doing different types of physical activity in a typical week. Please answer these questions even if you do not consider yourself to be a physically active person.  Think first about the time you spend doing work. Think of work as the things that you have to do such as paid or unpaid work, study/training, household chores, harvesting food/crops, fishing or hunting for food, seeking employment. *[Insert other examples if needed].* In answering the following questions 'vigorous-intensity activities' are activities that require hard physical effort and cause large increases in breathing or heart rate, 'moderate-intensity activities' are activities that require moderate physical effort and cause small increases in breathing or heart rate. | | | | | |
| **Question** | | **Response** | | **Code** | |
| 44 | Does your work involve vigorous-intensity activity that causes large increases in breathing or heart rate like *[carrying or lifting* *heavy loads, digging or construction work]*  for at least 10 minutes continuously? | Yes | 1 | E1 | |
|  |  | No | 2 |  |  |
| 45 | In a typical week, on how many days do you do vigorous-intensity activities as part of your work? | Number of days | └─┘ | E2 | |
| 46 | How much time do you spend doing vigorous-intensity activities at work on a typical day? | Hours : minutes | └─┴─┘: └─┴─┘  hrs mins | E3 (a-b) | |
| 47 | Does your work involve moderate-intensity activity, that causes small increases in breathing or heart rate such as brisk walking *[or carrying light loads]* for at least 10 minutes continuously?  *[INSERT EXAMPLES] (USE SHOWCARD)* | Yes | 1 | E4 | |
|  |  | No | 2 |  |  |
| 48 | In a typical week, on how many days do you do moderate-intensity activities as part of your work? | Number of days | └─┘ | E5 | |
| 49 | How much time do you spend doing moderate-intensity activities at work on a typical day? | Hours : minutes | └─┴─┘: └─┴─┘  hrs mins | E6 (a-b) | |
| **Travel to and from places** | | | | | |
| The next questions exclude the physical activities at work that you have already mentioned.  Now I would like to ask you about the usual way you travel to and from places. For example to work, for shopping, to market, to place of worship. *[Insert other examples if needed]* | | | | | |
| 50 | Do you walk or use a bicycle *(pedal cycle)* for at least 10 minutes continuously to get to and from places? | Yes | 1 | | E1a |
|  |  | No | 2 | |  |
| 51 | In a typical week, on how many days do you walk or bicycle for at least 10 minutes continuously to get to and from places? | Number of days | └─┘ | | E1b |
| 52 | How much time do you spend walking or bicycling for travel on a typical day? | Hours : minutes | └─┴─┘: └─┴─┘  hrs mins | | E1c (a-b) |
| 53 | How many hours do you usually watch TV? | Hours : minutes | └─┴─┘: └─┴─┘  hrs mins | | E1d |
| 54 | Do you Watch TV During your meal | Yes 1  No 2 |  | | E1e |
| 55 | On average, how many hours in night do you sleep? | More than 6 hours 1  Less than 6 hours 2 |  | | E1f |

|  | **CORE: History of Diabetes** |  |  |  |
| --- | --- | --- | --- | --- |
|  | **Question** | **Response** |  | **Code** |
| 56 | Have you ever had your blood sugar measured by a doctor or other health worker? | Yes  No | 1  2 |  |
| 57 | Have you ever been told by a doctor or other health worker that you have raised blood sugar or diabetes? | Yes  No | 1  2 | *S3* |
| 58 | If yes, How old are you ( Your age 1^st^ diagnosis) | Age (years)_______________ |  | *S3* |
| 59 | Are (were) any family members suffering from DM Disease? | Yes 1  No 2 |  | *S4* |
| 60 | Are you suffering from TB disease? | Yes 1  No 2 |  | *S5* |
| 61 | Can obese person get DM disease from being obesity: Not Sure? | Yes No |  |  |
| 62 | Does diabetes mellitus damage your organ | Yes No |  |  |
| 63 | Does diabetes mellitus disease seen with increased frequency with physical inactivity | Yes No |  | *S5* |

| Section 2 (**Step 2) Physical Measurements** | | | | |
| --- | --- | --- | --- | --- |
| **CORE: Height and Weight** | | | | |
| **Question** | | **Response** | | **Code** |
| 64 | Height | in Centimetres (cm) | └─┴─┴─┘. └─┘ | P1 |
| 65 | Weight | in Kilograms (kg) | └─┴─┴─┘.└─┘ | P2 |
| 66 | **For women:** Are you pregnant? | Yes | 1 | P3 |
|  |  | No | 2 |  |
| **CORE: Waist** | | | | |
| 67 | Waist circumference | in Centimetres (cm) | └─┴─┴─┘.└─┘ | W1 |
| 68 | Hip circumference | in Centimeters (cm) | └─┴─┴─┘.└─┘ | W2 |
| **CORE: Blood Pressure** | | | | |
| 69 | Cuff size used | Small | 1 | B1 |
|  |  | Medium | 2 |  |
|  |  | Large | 3 |  |
| 70 | Reading 1 | Systolic ( mmHg) | └─┴─┴─┘ | B2a |
|  |  | Diastolic (mmHg) | └─┴─┴─┘ | B2b |
|  | Reading 2 | Systolic ( mmHg) | └─┴─┴─┘ | B3a |
|  |  | Diastolic (mmHg) | └─┴─┴─┘ | B3b |
|  | Reading 3 | Systolic ( mmHg) | └─┴─┴─┘ | B4a |
|  |  | Diastolic (mmHg) | └─┴─┴─┘ | B4b |
| 71 | During the past two weeks, have you been treated for raised blood pressure with drugs (medication) prescribed by a doctor or other health worker? | Yes | 1 | B5 |
|  |  | No | 2 |  |

| **EXPANDED: Hip Circumference and Heart Rate** | | | | |
| --- | --- | --- | --- | --- |
| 72 | Heart Rate | | |  |
|  | Reading 1 | Beats per minute | └─┴─┴─┘ | H1a |
|  | Reading 2 | Beats per minute | └─┴─┴─┘ | H1b |
|  | Reading 3 | Beats per minute | └─┴─┴─┘ | H1c |

| Section **3 (Step 3) Biochemical Measurements** | | | | | | |
| --- | --- | --- | --- | --- | --- | --- |
| **CORE: Blood Glucose** | | | | | | |
| **Question** | | **Response** | | | **Code** | |
| 73 | During the past 12 hours have you had anything to eat or drink, other than water? | Yes | 1 | | B4 | |
|  |  | No | 2 | |  |  |
| 74 | Time of day blood specimen taken (24 hour clock) | Hours : minutes | └─┴─┘: └─┴─  hrs mins | | S5 | |
| 75 | Fasting blood glucose  *Choose accordingly: mmol/l or mg/dl* | mmol/l | └─┴─┘. └─┴─┘ | | S6 | |
|  |  | mg/dl | └─┴─┴─┘.└─┘ | |  |  |
| 76 | Today, have you taken insulin or other drugs (medication) that have been prescribed by a doctor or other health worker for raised blood glucose? | Yes | 1 | | S7 | |
|  |  | No | 2 | |  |  |
|  |  | No | 2 | |  |  |
| **CORE: Blood Lipids** | | | | | |  |
| 77 | Total cholesterol  *Choose accordingly: mmol/l or mg/dl* | mmol/l |  | L1 | |  |
|  |  | mg/dl |  |  |  |  |
| 78 | During the past two weeks, have you been treated for raised cholesterol with drugs (medication) prescribed by a doctor or other health worker? | Yes | 1 | L2 | |  |
|  |  | No | 2 |  |  |  |

| **EXPANDED: Triglycerides and LDL Cholesterol** | | | | |
| --- | --- | --- | --- | --- |
| 79 | Triglycerides  *Choose accordingly: mmol/l or mg/dl* | mmol/l |  | L3 |
|  |  | mg/dl |  |  |
| 80 | LDL Cholesterol  *Choose accordingly: mmol/l or mg/dl* | mmol/l |  | L4 |
|  |  | mg/dl |  |  |
| 81 | HDL: | Mmol/l |  |  |

**በአለም ጤና አጠባበቅ የስኳር በሽታን ጥቃት ምን ያህል እንደሆነና ተያያዥ የሆኑ ለበሽታው የሚያጋልጡ ነገሮችንለማጥናት የተዘጋጁ መጠይቆች ጎንደር ሰሜን ምእራብ ኢትዮዽያ**

የተሳታፊው መለያ ቁጥር ________________ ______________________________________ 2011

**THANK YOU VERY MUCH FOR BEING PART OF THIS STUDY! WE REALLY APPRECIATE YOUR WORKING WITH US IN THIS RESEARCH. YOU ARE HELPING US TO UNDERSTAND MORE ABOUT THE LIVES AND HEALTH OF PEOPLE**

**THANKS!**

ክቡራን ተሳታፊዎች

ይህ ጥናት በጎንደር ዩኒቨርስቲ ፣ አዲስ ኮንቲነንታል እና በጥናት አቅራቢው ተመራማሪ በጋራ የሚሰራ ነው

ጥናቱ በሁለት ቦታዎች በጎንደር ከተማና በዳባት ወረዳ ገጠራማ ቀበሌዎች ውስጥ ይካሄዳል::

ይህ ምርምር የፒኤችዲ ጥናት አካል ሲሆን አላማውም የስኳር በሽታ ጥቃት ምን ያህል እንደሆነና ተያያዥ የሆኑ ለበሽታው የሚያጋልጡ ነገሮችን በተሻለ መልክ ለመለየት በጎንደር ገጠርአማ አካባቢ እና በከተማ ባሉ ማህበረሰቦች ውስጥ በሰሜን ምእራብ ኢትዩጵያ የሚካሄድ ጥናት ነው:: እርሶወ በዚህ ጥናት እንዲሳተፉ ተመርጠዋል

የዚህ መጠየቅ አላማ የስኳር በሽታ ምን ያህል እንደሆነ ፣ ለስኳር በሽታ የሚያጋልጡ ነገሮች በሰውየው አኗኗር ፣ ባህሪ ፣ ማህበረዊ እና ሌላ ተመሳሳይ የሚያጋልጡ ነገሮችን መረጃ ለማሰባብ ነው ከመጠይቁ በተጨማሪ ቁመት፣ክብደት፣የወገብና ዳሌ ልክ፣የደም ግፊት መጠንና በደምዎት ውስጥ ያለው የስኳር መጠን የስኳር በሽታን ለመመርመር ይረዳ ዘንድ ይለካሎታል::

የሁሉም መረጃዎች ሚስጥር ይጠበቃል::

መረጃው ለምርምር አገልግሎት ብቻ ነው የሚውለው ፤ የማንም ሰው ስም በሚቀርበው ጥናታዊ ጽሁፍ ውስጥ አይገለጽም እርሶ በዚህ ጥናት ለመሳተፍ ከፈለጉ እባክዎት ከዚህ በታች በተዘጋጀው ቦታ ፈርመው ቀኑን ይጻፉ (አንዱ የቤተሰብ ፣ አባል ባለበት) ፡፡ እባክዎት ሁሉንም ጥያቄ ይመልሱ ለተለያዩ ጥያቂዎች እንዴት መመለስ እንዳለብወ የሚገልጽ ከእያንዳንዱ አርእስተ ጉዳይ መጀመሪያ ላይ ተጽፏል

እኔ ከዚህ በታች የፈረምኩት የፕሮጀክቱን አላማ የተረዳሁ ሲሆን እሱም የስኳር በሽታ ምን ያህል እንደሆነና ለስኳር በሽታ የሚያጋል ጡ ነገሮች በሰሜን ምእራብ ኢትዩጵያ በጎንደር ከተማና በዳባት ገጠርአማ ሕብረተሰብ የሚካሄድ መሆኑ እንዲሁም የጥናቱ ተሳታፊ ለመሆንና በጥናቱ ለመካተት የጥናቱ አቅራቢ እንደገለጡልኝ ተስማምቻለሁ ለዚህም ጥያዌዎችን ለመሙላትና ለላብራቶሪ ምርመራ ደም ለመስጠት ተስማምቻለሁ

ስም _____________________________________________________ ፊርማ ___________________________________

የተሳታፊ መለያ ቁጥር ____________________

ቀበሌ _________________________

ወረዳ/ከተማ _____________________

የተሳታፊው መለያ ቁጥር ______

| ቁጥር | ቦታና ቀን | መልስ | መለያ ቁጥር |
| --- | --- | --- | --- |
| 1 | የአካባቢው / የመንደሩ/ መለያ |  | D1 |
| 2 | የአካባቢው የቀበሌ/ቀጠና ስም |  | D2 |
| 3 | የጠያቂው መለያ ቁጥር |  | D3 |
| 4 | ፎርሙ የተሞላበት ቀን | ቀን _____________ ወር __________ ዓም ______________ | D4 |
| 5 | የቤት ቁጥር |  | D5 |
| 6 | ቃለ መጠይቅ የተደረገበት ቋንቋ | 1. እንግሊዘኛ 2. አማርኛ 3. ሌላ | D6 |
| 7 | የትውልድ ቦታ |  | D7 |
| 8 | ሀይማኖት / እምነት | 1. ኦርቶዶክስ 2. ሙስሊም 3. ሌላ | D8 |
| 9 | ቃለ መጠይቅ የተደረገበት ሰዓት | ሰዓት ደቂቃ | D9 |
| 10 | ጾታ | 1. ወንድ  2. ሴት | D10 |
| 11 | እድሜዎ ስንት ነው | ዓመት | D12 |
| 12 | የትምህርት ደረጃ | 1. መደበኛ ትምህርት ያልተማረ/ ያልተማረች   88. ለመናገር ፈቃደኛ አይደለሁም | D13 |
| 13 | ብሔርዎት ምንድነው | 1. አማራ  2. ትግሬ 3. ኦሮሞ  4. ሌላ ካለ ይጠቀስ  5. ለመናገር ፈቃደኛ አይደለሁም | D14 |
| 14 | የጋብቻ ሁኔታ | 1. ያላገባ/ች 2. ባለትዳር 3. የተለያየ/ች 4. የተፋታ/ች  5. የሞተበት/የሞተባት  88. ለመናገር ፈቃደኛ አይደለሁም | D15 |
| 15 | ባለፉት 12 ወራት ያለዎት የስራ ሁኔታ | 1. የመንግስት ተቀጣሪ  2. የግል ድርጅት ተቀጣሪ  3. የግል ስራ  4. የማይከፈል ስራ  5. ተማሪ  6. የቤት ሠራተኛ  7. ጡረታ  8. ስራ የሌለው (መስራት ይችላል/ ትችላለች)  9. ስራ የሌለው (መስራት አይችልም )  88. መልስ መስጠት ፈቃደኛ አይደለሁም | D16 |

**አሁን ከጤናዎት ጋር የተያያዘ አንድአንድ ጥያቄዎች ልጠይቅዎት ነው፡፡ እነዚህም ስለ ማጨስ፤አልኮል መጠጣት፤ፍራፍሬና አትክልትን መመገብ እና የሰውነት እንቅስቃሴ ማድረግ፡፡ እስኪ ከማጨስ እንጀምር**

| ቁጥር | ቦታና ቀን | መልስ | መለያ ቁጥር |
| --- | --- | --- | --- |
| 16 | የትምባሆ ዉጤቶችን አሁን ያጨሳሉ፣ | 1. አዎ 2. አልጠቀምም | T1 |
| 17 | የትምባሆ ዉጤቶችን በየቀኑ ያጨሳሉ፣ | 1. አዎ 2. አልጠቀምም | T2 |
| 18 | በየቀኑ ማጨስ ሲጀምሩ ዕድሜዎት ስንት ነበር፤ | ዓመት  77. አላውቅም | T3 |
| 19 | በየቀኑ ማጨስ ሲያቆሙ ዕድሜዎት ስንት ነበር፤ | ዓመት  77. አላውቅም | T4 |
| 20 | በአሁኑ ሰዓት የማይጨሱ ትምባሆዎችን ይጠቀማሉ (ለምሳሌ ባፍንጫ የሚሳብ) | 1. እጠቀማለሁ  2. አልጠቀምም | T5 |
| 21 | የሚታኘኩ የትምባሆ ወጤቶችንስ የጠቀማሉ | 1. እጠቀማለሁ  2. አልጠቀምም | T6 |
| 22 | በየቀኑ በአማካይ ስንት ሲጋራ ይጠቀማሉ | ቁጥር | T7 |
| 23 | ባለፉት 7 ቀናት ውስጥ እርስዎ ባሉበት ቤት ሌላ ሰው ለስንት ቀን አጨሱ | ቀን  77. አላውቅም | T8 |
| 24 | ባለፉት 7 ቀናት ውስጥ ሌላ ሰው ለስንት ቀናት በሚሰሩበት አካባቢ (ሕንጻ ቢሮ) እርሶ ባሉበት አጨሰ | ቀን  77. አላውቅም | T9 |

**የሚቀጥለው ጥያቄ ስለ አልኮል አጠቃቀም ነው**

| ቁጥር | ቦታና ቀን | መልስ | መለያ ቁጥር |
| --- | --- | --- | --- |
| 25 | የአልኮል መጠጥ ጠጥተው ያውቃሉን (ወይን ፣ ቢራ ፣ ጠላ ጠጅ ፣ አረቄ እና የመሳሰሉት) | 1. አዎ 2. አልጠቀምም | A1 |
| 26 | ባለፉት 12 ወራት ውስጥ አልኮል ጠጥተው ነበር | 1. አዎ 2. አልጠቀምም | A2 |
| 27 | ባለፉት 12 ወራት ውስጥ በየስንት ጊዜ ቢያነስ አነድ አልኮል ጠጥተዋል | 1. በየቀኑ 2. 5-6 ቀናት በሳምንት ውስጥ  3. 1-4 ቀናት በሳምነት ውስጥ 4. 1-3 ቀናት በወር 5. ከ 1 ቀን በታች በወር | A3 |
| 28 | ባለፉት 30 ቀናት ውስጥ አልኮል ጠጥተው ያውቃሉ | 1. አዎ 2. አልጠቀምም | A4 |
| 29 | ባለፉት 30 ቀናት ውስጥ ስንት አጋጣሚዎች ነበሩ ቢያንስ አንድ አልኮል ለመጠጣት | በቁጥር  77. አላውቀውም | A5 |
| 30 | ባለፉት 30 ቀናት ውስጥ ደረጃቸውን የጠበቁ አልኮሎች (ለወንዶች 5 ወይም ከዛ በላይ ለሴቶች 4 ወይም ከዛ በላይ) በአማካይ በአንድ አጋጣሚ (ግብዣ) ስንት ጠጥተዋል | በቁጥር  77. አላውቀውም | A6 |

**አልኮል መጠቀም**

| ቁጥር | ቦታና ቀን | መልስ | መለያ ቁጥር |
| --- | --- | --- | --- |
| 31 | ባለፉት 30 ቀናት ውስጥ መቅሰስን ሳይጨምር አልኮል ከምግብ ጋር የወሰዱት ለስንት ጊዜ ነው | 1. ሁልጊዜ ከምግብ ጋር ነው  2. አንዳንድ ጊዜ ከምግብ ጋር ነው  3. አልፎ አልፎ ከምግብ ጋር ነው  4. በጭራሽ ከምግብ ጋር አይደለም | A7 |
| 32 | ባለፉት ሰባት ቀናት ውስጥ ስንት አልኮሎች በየቀኑ ጠጡ | ሰኞ _____________________  ማክሰኞ _________________  ዕሮብ __________________  ሀሙስ ______________________  አርብ __________________  ቅዳሜ _____________________  እሁድ_____________________ | A1a  A1b  A1c  A1d  A1e  A1f A1G |

**የሚቀጥለው ስለ አትክልት ፍራፍሬ አጠቃቀምዎ ለማወቅ የቀረበ ሲሆን ለዚህም ምስሎችን የሚያሳየውን ካርድ እየተመለከቱ እንዲመልሱልኝ እጠይቃለሁ እየንዳንዱ ምስል የሚቀርበውን ብዛት ያመለክታል አመቱ ውስጥ አትክልት እና ፍራፍሬ የሚያዘወትሩበትን ሳምንት በማስታወስ ይመልሱ፡፡**

| ቁጥር | ቦታና ቀን | መልስ | መለያ ቁጥር |
| --- | --- | --- | --- |
| 33 | አዘውትረው በሚመገቡበት ሳምንት ስንት ቀን ፍ**ራ**ፍሬ ይበላሉ | የቀን ቁጥር  77. ምንም | N1 |
| 34 | አትክልት በሳምንት ስንት ቀን ይመገባሉ | የቀን ቁጥር  77. ምንም | N2 |
| 35 | በቤትዎ ውስጥ ምግብ ለማዘጋጀት ምን አይነት ዘይት ወይም ቅባት ይጠቀማሉ | 1. የአትክልት ዘይት  2. የቅባት እህል ጥራ ጥሬ (ኑግ ሰሊጥ  ወዘተ) 3. ቅቤ ወይም የተጣራ ቅቤ 4. ማርጋሪን 5. ሌላ ከሆነ ይገለጥ  6.በተለየ የምጠቀመው የለም (የተገኘውን  እጠቀማለሁ) 7. ምንም አልጠቀምም 8. አላውቅም | N3 |
| 36 | ባለፉት 30 ቀናት ውስጥ ስንት አጋጣሚዎች ነበሩ ስጋን በምግብነት የተመገቡት | 1. ምንም አልጠቀምም 2. በቀን 3. በሳምነት 4. በወር | N4 |
| 37 | ይጾማሉ | 1. አዎ 2. አይደለም | N5 |
| 38 | የሚጾሙ ከሆነ በቀን ለምን ያህል ሰዓት ይጾማሉ | ሰዓት ደቂቃ | N6 |
| 39 | በአመትስ ለስንት ቀንኖች ይጾማሉ | ቀን | N7 |
| 40 | በጾም ግዜ የሚመገቡት የምግብ አይነት | 1. የእንስሳት ተዋፅኦ (ስጋ፣ወተት፣አሳ) 2. ጥራጥሬ እና አትክልት 3. ሁሉንም ያካተተ | N8 |
| 41 | እንጀራ ይመገባሉ | 1. አዎ 2. አልጠቀምም | N9 |
| 42 | በቀን ለስንት ግዜ | 1. አንድ 2. ሁለት 3. ሶስት | N10 |
| 43 | እንጀራው ከምን አይነት የእህል ዘር ይዘጋጃል | 1. ጤፍ 2. ዳጉሳ 3. ሩዝ 4. ቅልቅል   ሌላ ይገለጥ | N11 |

በሚቀጥለው የምጠይቆት በሳምንት ውስጥ ለምን ያህል ጊዜ የተለያዩ የአካል እንቅስቃሴዎቸን እንደሚያደርጉ ነው እባክዎት ይህን ጥያቄ አካላዊ እንቅስቃሴ የማላደርግ ነኝ ብለውም ቢያስቡ እንኳን ጥየቄውን ይመልሱ መጀመሪያ ስራ በመስራት የሚሳልፉበትን ጊዜ ያስቡ ስራዎን በክፍያ ወይመ ያለክፍያ ቢሰሩም ጥናት/ስልጠና ፣ የቤት ውስጥ ስራ ምግቦችን /ስብሎችን መሰብሰብ ፣ አሳ ማጥመድ ወይም ምግብ ማፈላለግ ስራ መፈለግ (ተጨማሪ ምሳሌ ስጥ ካስፈለገ) ይህን ጥያቄ ሲመልሱ ከባድ አካላዊ ዕንቀወስቃሴ የሚባው እንቅስቃሴውን ለመስራት ጠንካራ አካላዊ ትረት የሚጠይቅ እንዲሁም የትንፋሺን አተነፋፈስ የሚጨምር ሲሆን መካከለኛ አካላዊ እንቅስቃሴ የሚባለው መካከለኛ አካላዊ ጥረት የሚጠይቅ እንዲሁም ትንፋሾዎን ትንሽ የሚጨመር ነው

| ቁጥር | ቦታና ቀን | መልስ | መለያ ቁጥር |
| --- | --- | --- | --- |
| 44 | ስራዎት ከባድ ጉልበት የሚጠይቅና ትንፋሾዎንና የልብ ምችን የሚጨምር ነው (ለምሳሌ ከባድ እቃ መሸከም መቆፈር እና የሕንጻ ስራ) | 1. አዎ 2. አይደለም | E1 |
| 45 | በሳምንት ውስጥ ከበድ ጉልበት የሚጠይቅ ስራ ለስንት ቀን ይሰራሉ | ቁጥር | E2 |
| 46 | በቀን ውስጥ ከባድ የጉልበት ስራ በመስራት ስንት ሰዓት ያጠፋሉ | ሰዓት ደቂቃ | E3 |
| 47 | ስራዎት መካከለኛ ጉልበት የሚጠይቅ እንዱም ትንፋሾዎንና የልብ ምቶችን በትንሹ የሚጨምር ነው( ፈጣን እርምጃ ወይም ቀለል ያለ ሸክም መሸከም ቢያንስ ለ 10 ደቂቃ ተከታታይ | 1. አዎ 2. አይደለም | E4 |
| 48 | በሳምንት ውስጥ መካከለኛ ጉልበት የሚጠይቅ ስራ ለስንት ቀን ይሰራሉ | ቁጥር | E5 |
| 49 | በቀን ውስጥ መካከለኛ ጉልበት የሚጠይቅ ስራ በመስራት ስንት ሰዓት ያጠፋሉ | ሰዓት ደቂቃ | E6 |

**የሚቀጥለው ጥያቄ እርሶ የነገሩንና በስራዎት ላይ ያለውን የሰውነት እንቅስቃሴ የሚያካትት ሳይሆን ከቦታ ቦታ የሚያደርጉትን እንቅስቃሴ የሚመለከት ነው፡፡**

| ቁጥር | ቦታና ቀን | መልስ | መለያ ቁጥር |
| --- | --- | --- | --- |
| 50 | ከቦታ ቦታ ለመዘዋወር በእግሮት ይሄዳሉ ወይስ ብስክሌት ሞተር ብስክሌት ቢያንስ ለ10 ደቂቃ ይጠቀማሉ | 1. አዎ 2. አልጠቀምም | E1a |
| 51 | በሳምንት ውስጥ ከቦታ ቦታ ለመዘዋወር ስንት ቀን በእግሮት ወይም በብስክሊት ካለማቋረጥ ለ 10 ደቂቃ ተጉዘዋል | ቁጥር | E1b |
| 52 | በቀን ውስት በእግሮት ወይም በብስክሌት በመጓዝ ስንት ሰአት ያሳልፋሉ (ያጠፋሉ) | ሰዓት ደቂቃ | E1c |
| 53 | ለምን ያህል ሰዓት ቲቪ ያያሉ | ሰዓት ደቂቃ | E1d |
| 54 | በምግብ ሰዓት ቲቪ ይመለከታሉ | 1. አዎ 2. አይደለም | E1e |
| 55 | ማታ ማታ በአማካይ ምን ያህል ሰዓት ይተኛሉ | ከ 6 ሰዓት በላይ  ከ 6 ሰዓት በታች | E1f |

**በደም ውስጥ የስኳር መጠን በተመለከተ**

| ቁጥር | ቦታና ቀን | መልስ | መለያ ቁጥር |
| --- | --- | --- | --- |
| 56 | ደምዎት ውስጥ ያው የስኳር መጠን በሀኪም ወይም በሌላ ጤና ባለሙያ ተለክቶ ያውቃሉ | 1. አዎ 2. አይደለም | S1 |
| 57 | በሀኪም ወይም በሌላ ጤና ባለሙያ በደምዎት ውስጥ ያለው የስኳር መጠን ጨምሯል ወይም የስኳር በሽታ አለበት ተብሎ ተነግሮት ያውቃሉ | 1. አዎ 2. አይደለም | S2 |
| 58 | ከሆነ በተነገረዎት ግዜ እድሜዎ ስንት ነበር | ዓመት | S3 |
| 59 | ከቤተሰብዎ ውስጥ በስኳር በሽታ ህመም ያለበት ሰው አለ | 1. አዎ  2. አይደለም | S4 |
| 60 | በሳንባ ነቀርሳ (ቲቢ) በሽታ ተይዘው ያውቃሉ | 1. አዎ   1. አይደለም | S5 |
| 61 | ወፍራም የሆነ ሰው በመወፈሩ ምክንያት የስኳር በ.ሺታ ሊይዘው ይችላል? | 1. አዎ  2. አይደለም | S6 |
| 62 | የስኳር በሺታ የሰውነት አካልዎን ይጎዳል ብለው ያስባሉ | 1. አዎ  2. አይደለም | S7 |
| 63 | የስኳር በሺታ አዘውትሮ የሰውነት እንቅስቃሴ በማለማድረግ ሊከሰት ይችላል ብለው ያስባሉ | 1. አዎ  2. አይደለም | S8 |

**የአካል መጠን** የተሳታፊው መለያ ቁጥር ______

| ቁጥር | ቦታና ቀን | መልስ | መለያ ቁጥር |
| --- | --- | --- | --- |
| 64 | ቁመት | ሴ.ሚ. | P1 |
| 65 | ክብደት | ኪ.ግ. | P2 |
| 66 | ለሴቶች - ነፍሰጡር ነዎትን | 1. አዎ 2. አይደለም | P3 |
| 67 | የወገብ ልክ | ሴ.ሚ. | W1 |
| 68 | የዳሌ ልክ | በሴንቲ ሜትር | W2 |

**የደም ግፊት**

| ቁጥር | ቦታና ቀን | መልስ | መለያ ቁጥር |
| --- | --- | --- | --- |
| 69 | የተጠቀምንበት መሳሪያ (ለደም ግፊት) | 1. ትንሽ  2. መካከለኛ 3. ትልቅ | B1 |
| 70 | የመጀመሪያው ንባብ (ውጤት) | ሲያስቶሊክ (mmHg)  ዲያስቶሊክ (mmHg) | B2a  B2b |
|  | የሁለተኛው ንባብ (ውጤት) | ሲያስቶሊክ (mmHg)  ዲያስቶሊክ (mmHg) | B3a  B3b |
|  | የሶስተኛው ንባብ (ውጤት) | ሲያስቶሊክ (mmHg)  ዲያስቶሊክ (mmHg) | B4a B4b |
| 71 | ባለፉት ሁለት ሳምንታት ውስጥ ለደም ግፊት መጨመር በሀኪም ወይም በሌላ ጤና ባለሙያ መድሀኒት ታዞልሕ ነበር | 1. አዎ 2. አይደለም | B5 |

**የልብ ምት**

| ቁጥር | ቦታና ቀን | መልስ | መለያ ቁጥር |
| --- | --- | --- | --- |
|  |  |  |  |
| 72 | የልብ ምት |  |  |
|  | የመጀመሪያው ውጤት | ምት በደቂቃ | H1a |
|  | የሁለተኛው ውጤት | ምት በደቂቃ | H1b |
|  | የሶስተኛው ውጤት | ምት በደቂቃ | H1c |

**በደም ውስጥ የስኳር መጠን**

| ቁጥር | ቦታና ቀን | መልስ | መለያ ቁጥር |
| --- | --- | --- | --- |
| 73 | ባለፉት 12 ሰዓታት ውስጥ ከውሃ ሌላ የጠጡት ወይም የተመገቡት ምግብ ወይም መጠጥ አለ | 1. አዎ 2. አይደለም | S4 |
| 74 | የደም ናሙና የተወሰደበት ሰዓት | ሰዓት ደቂቃ | S5 |
| 75 | በደምዎት ውስጥ ያለ አጠቃላይ የስኳር መጠን | mg/dl | S6 |
| 76 | ዛሬ ደመዎ ውስጥ ለጨመረ ስኳር በመርፌ የሚሰጥ (ኢንሱሊን)ወይም ሌላ የሚዋጥ መድሃኒት በሃኪም ወይም በሌላ ጤና ባለሙያ የታዘዘሎትን ወስደዋል | 1. አዎ 2. አይደለም | S7 |

| **CORE: Blood Lipids profile** | | | | |
| --- | --- | --- | --- | --- |
| 77 | Total cholesterol  *Choose accordingly: mmol/l or mg/dl* | mmol/l |  | L1 |
|  |  | mg/dl |  |  |
| 78 | During the past two weeks, have you been treated for raised cholesterol with drugs (medication) prescribed by a doctor or other health worker? | Yes | 1 | L2 |
|  |  | No | 2 |  |

| **EXPANDED: Triglycerides and LDL Cholesterol** | | | | |
| --- | --- | --- | --- | --- |
| 79 | Triglycerides  *Choose accordingly: mmol/l or mg/dl* | mmol/l |  | L3 |
|  |  | mg/dl |  |  |
| 80 | LDL Cholesterol  *Choose accordingly: mmol/l or mg/dl* | mmol/l |  | L4 |
|  |  | mg/dl |  |  |
| 81 | HDL: | Mmol/l |  |  |

**እናመሰግናለን**
