# Supplementary material for: Magnitude of metabolic syndrome in Gondar town, Northwest Ethiopia: A community-based cross-sectional study
Source: PLoS One. 2021 Oct 7;16(10):e0257306. doi: 10.1371/journal.pone.0257306 (PMC8496848; doi:10.1371/journal.pone.0257306)
Supplement: Attachment — Submitted filename: New Microsoft Word Document (2).docx [file pone.0257306.s003.docx]

Abstract

Background: Metabolic Syndrome (Mets) is becoming a major public health problem globally;

It is clear that the burden of Mets and its effects on Non- communicable diseases is rising rapidly.

In Ethiopia studies done so far have shown a large disparity in magnitude and were mainly institution-based studies. Therefore, this study aims to establish baseline community-based data on Mets using Adult Treatment Panel III (ATP III and IDF) which is imperative to utilize developing and strengthening national NCD prevention and control programs.

Methods: This study was conducted in Gondar city Northwest Ethiopia. This study employs a community-based cross-sectional study design among 3,227 individuals age greater than or equal to 18 years older. Data were collected using the WHO stepwise tool. The prevalence estimation was made along with a 95% confidence interval (CI). The Kappa statistic was used to analyze the statistical agreement between ATP III and IDF definitions of the Mets. Stratified analysis was also performed for description and analysis components using ATP III and IDF as an outcome.

Result: Of the total study participant 3059 (94.8%) were included in the final analysis and 52.5% were female. The mean (±SD) age of the study participant was 40.8 years (16.2 ±SD). The overall prevalence of Mets using ATP III was 11.2% [95%CI: 10.1, 12.3] and that of IDF was 11.9% [95%CI: 10.8, 13.2]. The sex-specific proportion was high in females than males irrespective of the criteria. The prevalence of high total cholesterol was 22.4%, high LDL 13.8%, Low HDL 34.2%, and that of high triglyceride 22.2%. The overall level of agreement between ATP III and IDF prevalence was 91.7% and the Kappa statistics was 0.594. Increase in age, low-density lipoprotein cholesterol, Body mass index, being female, born in an urban area, consumed an Alcoholic drink in the preceding 30 days and non-fasting practice was significantly associated with Mets.

Conclusion and recommendation: There was a high prevalence of Mets among females than males irrespective of Mets diagnostic criteria. This also shows good agreement between ATP III and IDF. We also observed a high proportion of the study participant to have low HDL protein.

Low-density lipoprotein cholesterol, Body mass index, being female, being of urban birthplace, frequent alcoholic consumption in the last 30 days, and frequently fasting are factors associated with high metabolic syndrome. Hence, awareness campaigns and nutrition education intervention should be undertaken to promote health behavioral practice.

Keywords: Mets, IDF, ATP III criteria, metabolic syndrome, High cholesterol

Background

Metabolic syndrome (Mets) refers to a condition that inhabits three of the five following medical conditions which include central obesity, decreased high-density lipoprotein cholesterol, elevated triglycerides, elevated blood pressures, and hyperglycemia [1,2]. Becoming a major challenge to global development, it is clear that the burden of Mets and its effects on Non- communicable diseases is rising rapidly [2]. With that being said, it could be observed that the low- and middle-income countries of the world are being the most affected by these diseases which will have substantial social, economic, and health consequences [3-5].

Despite their rapid growth and inequitable distribution, much of the human and social impact caused each year by NCD-related deaths could be averted through well-understood, cost-effective and feasible interventions [6]. Through clear, cost-effective, and achievable interventions most of the human and social bearing caused yearly by NCD-related deaths could be prevented. On top of the direct health care costs, the economic impact of early death and disability is potentially devastating in developing countries. Internationally the prevalence is different among regions, one of the lowest prevalence was observed in China among children (0.7%) (0.7%) [7] And the higher prevalence observed in Indus Hospital, Karachi, Pakistan (97.5%) [8] And in Palestinian refugees among obese and overweight participant being 69.4% [1].

Fast-paced urbanization and modernization changes related to unhealthy lifestyles coupled with climatic changes give rise to intermediate-risk factors such as Mets, unfavorable lipid profiles, and obesity; which in turn weaken individuals to the cardiovascular disease [9, 10]. With the current rapid urbanization people in developing countries are increasingly eating processed foods and are being targeted by marketing for tobacco, alcohol, and junk food, while, inevitably, availability of these products increases Mets. Moreover, currently, more and more labor-saving devices that keep people increasingly indoors are being created which could be an explanation for the increases in obesity and CVD [11, 12].

Ethiopia is facing a dual burden, on the one hand with a high number of infectious diseases and on the other an increasing degree of NCDs. Ultimately this led to an update of the government's strategic plan in such a way that it was forced to combat NCDs in the country [13]. This burden on multiple fronts, which is far beyond what is experienced in developed countries, imposes a demand that the health service resources of Ethiopia could hardly accommodate. Contrary to this very little evidence exists on the epidemiology of metabolic syndrome at a community level in Ethiopia and yet no adequate attention has been given to the epidemic and care management with the other competing groups of diseases.

Studies done so far has shown a large disparity in the magnitude of Mets, in Ethiopia, one of the studies done in 2015 among 9 regions and two city administrations including Addis Ababa, showed a prevalence of Mets of 4.8%. Another population-based survey conducted in Jimma from 2008– 2009 showed 10.7% and a recent study in Addis Ababa showed a prevalence of 20.3% [3, 14, and 15].

Besides this, most of the Mets studies in Ethiopia were institution-based and urban-focused.

Moreover, to fully comprehend the standing of Dyslipidemia in Ethiopia population-centered epidemiological documented information is necessary and essential. Studies done until now seemed to have gaps in terms of lifestyle and anthropological perspectives which are needed to explain the reasons, deterrence, and control of metabolic syndrome, especially in Ethiopia, where health outcomes are highly dependent on cultural variables [16] [17]. Therefore, an establishment of baseline data on Mets using Adult Treatment Panel III (ATP III and IDF) is imperative to utilize developing and strengthening national NCD prevention and control programs.

Method and Material

Study context

This study was conducted in Gondar town Northwest Ethiopia, located in East Africa. Ethiopia is Africa's second-most populous country with an estimated population of 109,224,414 in 2018, according to the 2019 revision of the World Population Prospects, of which about 18% live in urban areas. Gondar is a densely populated historical city of the region with an estimated urban population of 356,290.

Study Design

This study implemented a community-based cross-sectional study design.

Study population

All individuals of age greater than or equal to 18 years who reside in Gondar city for at least six months were included. All permanent residents in the study area aged 18 years and older were eligible to participate in the study.

Sample size and Sampling

The sample size for the study was determined by assuming 50% prevalence with a 95% confidence interval and a 5% margin of error. The study also considered a 5% non-response rate.

After having the minimum sample size stratification of the sample for age and sex was done (four age categories multiplied by two (for male and female), accordingly, the calculated sample size was 3227 individuals. A detail of the method was published elsewhere [18].

A two-stage random sampling strategy was used to select study administrative Kebele. First, four Districts (smallest administrative units, known as kebeles in Ethiopia) were selected using simple random sampling from the 12 administrative kebeles. Secondly, households were selected within each district using the systematic random sampling technique. Finally, an individual from the household was selected using a random lottery method. All the individuals in each household were given an identification number by the data collector.

Data collection

Data were collected by trained field workers that included local community enumerators, laboratory technicians, and nurses by going house-to-house. To ensure the quality of the interview, data collectors were trained by the principal investigator, and later on, random checks were carried out by field supervisors and the principal investigator. Data was collected by interviewing eligible subjects using a structured questionnaire. House-to-house data collection was performed by trained Laboratory technicians and Health extension nurses.

Measurements

The WHO and IDA criterion was used to classify hypertension with systolic blood pressure (SBP) of ≥140mmHg and /or diastolic blood pressure (DBP) ≥ 90 mmHg or known hypertensive patients on treatment. Anthropometric measurements were taken using standardized techniques and calibrated equipment. Blood pressure (BP) was measured using a digital measuring device with participants sitting after resting for at least five minutes. Finally, biochemical tests (Impaired fasting blood glucose levels (IFG) was between 110 and 125 mg/dl, triglyceride, LDL, HDL, and total cholesterol test) were carried out. Blood samples were collected from each participant by a trained laboratory technician following aseptic techniques. The blood samples were taken to the hospital laboratory for chemistry analyses. Waist circumference (WC) was categorized as low risk if it was less than 94 cm for men, and less than 80 cm for women; high risk if it was 94 cm or more for men, and 80 cm or more for women. Body mass index (BMI) was calculated as the ratio of weight in kilograms to the square of height in meters. BMI was used to define underweight (BMI < 18.5), normal (18.5 ≤ BMI < 25.0), overweight (25.0 ≤ BMI < 30.0), and obese (BMI ≥ 30) adults. Current alcohol consumption was assessed by asking participants to respond by ticking "Yes /No" to the question, "have you consumed any alcoholic drink, such as beer, wine, Tela, Tej, local Areki, fermented cider in the last 30 days?". Data about current smoking was found out by asking participants to respond in the same to the question, "Do you currently smoke any tobacco products, such as cigarettes?" Moderate physical activity was considered as "Yes" for those participants who walked at least for 10 minutes continuously daily [18].

Dependent variable

To measure the dependent variable we use IDF Definition: By the IDF criteria, subjects were classified as having Mets if participants had abdominal obesity (defined as waist circumference of ≥94 cm for men and ≥80 cm women) plus two of any of the following risk factors: (1) raised TG level (≥150 mg/dL); (2) reduced HDLC (<40 mg/dL in males and <50mg/dL in females); (3) raised blood pressure (systolic BP ≥130 or diastolic BP ≥85mmHg) or treatment of previously diagnosed hypertension; (4) raised FG (≥100mg/dL). Modified NCEP-ATP III Definition: Under the ATP III criteria, subjects were classified as having Mets if participants had three or more of the following risk factors: (1) abdominal obesity (waist circumference >102 cm in males and >88 cm in females); (2) hypertriglyceridemia (TG ≥150mg/dL); (3) reduced HDL-c (<40 mg/dL in males and <50mg/dL in females); (4) high BP (≥130/85mmHg); and (5) FG (≥100mg/dL).

Data management and analysis

Double data entry procedures were done using the EPI Info 7 statistical software. Distributive statistics were performed using tables and graphs. The prevalence estimation was made along with a 95% confidence interval (CI). The Kappa (kappa measure of interrater agreement Scale reliability coefficient) statistic was used to analyze the statistical agreement between ATP III and IDF definitions of metabolic syndrome. Stratified analysis was also performed to describe Mets using ATP III and IDF for the multivariate analysis, independent variables were selected based on a cut of point p-value <0.20 was included for the multivariable model. Binary logistic regression was applied to identify factors associated with Mets. A p-value of < 0.20 was used as criteria to include it in the multivariable logistic regression model to control confounding effects, and the results were considered statistically significant at a P-value ≤ of 0.05. Detail of the procedures is published elsewhere [18].

Ethical Statement.

Ethical clearance was obtained from the University of Gondar ethical review board. Subjects who volunteered to participate in the study were included after signing a written informed consent.

They were informed of their rights to withdraw from the study at any stage. For the sake of privacy and confidentiality, no personal identifiers such as names were collected. Result of the total study participant, 3059 (94.8%) were included in the final analysis and 52.5% were female. The mean (±SD) age of the study participant was 40.8 years (16.2 ±SD), the majority (53.12) of them did not attend formal education and 1728 (56.5%) of the study participants were married.

Only 520 (17%) were government and private employees. Socio-demographic characteristics of the study participants are presented in Table 1.

The overall prevalence of Mets using ATP III was 11.2% [95%CI: 10.1, 12.3] and that of IDF was 11.9% [95%CI: 10.8, 13.2]. The sex-specific proportion was high in females (16.3% than males (5.2%) using ATP III and using IDF was 16.8% in females and 6.3% in the male study population.

The prevalence of Mets increased with increasing age (1.9% in the age group of 18 to 24 years to 20.2% in the age group of 45 to 54 years). The prevalence of high total cholesterol in the study population was 22.4%, high LDL 13.8%, Low HDL 34.2%, and high waist circumference 26.9%, and that of high triglyceride 22.2%. Detail of the Mets profile is presented in Figures 1 and 2.

The proportion of Mets was slightly higher among diabetic subjects (37.2%) using both IDF and ATP III criteria compared to its proportion among subjects with high blood pressure 27.4% using ATP III and 26.5% using IDF criteria.

The distribution of high triglyceride, high total cholesterol, and high LDL cholesterol by age and sex are presented in Figures 2, 3, and 4.

The overall level of agreement between ATP III and IDF prevalence was 91.7%, which does not account for the chance. On the other hand, the Kappa statistics was 0.594 with the agreement deference being due to chance. In terms of sex, Kappa is better among females (0.589) than males (0.507).

The risk factor for cardiovascular disease, in general, is presented in supplement table 2.Variables included in the univariate analysis were age, sex, resident, religion, education status, marital status, main work type current smoking, current alcohol intake, vegetable and fruit intake, fasting habit, doing moderate exercise, history of TB, but only those whose p-value less than o.2 were included in the final model. The multivariable logistic regression model showed that every unit increase in age by one year will increase Mets by 1% [AOR=1%; 95%CI: 1.01, 1.02]. A unit increase in low-density lipoprotein cholesterol in milligram /deciliter will increase Mets by 1%,

[AOR= 1%; 95%CI: 1.00, 1.01]. Similarly, A unit increase in Body mass index of weight per cm2 will increase Metabolic Syndrome by 18% [AOR= 18%; 95%CI: 1.15, 1.22]. The occurrence of Mets was more than 2 times high among females than males [AOR=2.40; 95% CI: 1.82, 3.18]. The occurrence of Mets is 50% higher among people who were born in an urban area than their rural counterpart [AOR=1.50; 95%CI: 1.18, 1.93]. The odds of Mets were about 50% higher among subjects who had consumed an alcoholic drink in the preceding 30 days than those who didn't consume an alcoholic drink in the preceding 30 days [AOR=1.57; 95%CI: 1.22, 2.02]. The occurrence of Mets was more than 30% higher among non-fasting people than frequently fasting religious people at the time of fast (regardless of the classification) AOR= 1.42; 95%CI: 1.08, 1.87] (see Table 3).

Discussion

This study is one of the very few studies to assess Mets in the community, and we have uncovered a high prevalence of Mets among females rather than males irrespective of Mets diagnostic criteria. The overall level of agreement between ATP III and IDF prevalence was high (91.7%). In terms of sex, Kappa is better among females (0.589) than males (0.507) (91.7%) [19]. the finding is in line with a previous study done in Ethiopia (K 0.54) [20] and in Gahan [21]. We also observed a high proportion of the study participant to have low HDL protein. The proportion of Mets was slightly higher among subjects with high fasting blood glucose than those with high blood pressure cases. Low-density lipoprotein cholesterol, Body mass index, being females, urban birthplace, frequent alcoholic consumption for the last 30 days, and frequently fasting are factors associated with high metabolic syndrome irrespective of the criteria used.

This study revealed that there is a considerable rise in the prevalence of Mets in the community. This finding is consistent with a study done in Addis Ababa Ethiopia [22] and slightly higher than the findings in community-based studies done in an Indian rural community and residents of MizanAman Ethiopia [14, 22, 23]. The prevalence of Mets in this study is lower compared to findings of studies done in Ghana[21] and in rural Uganda [21]; however, those studies were conducted among the non-diabetic population. Similarly, the prevalence of Mets in the study was lower than reports in Jimma, South West Ethiopia and that of the University of Gondar Hospital [17,24]; but those two studies were conducted among individuals with mental health problems. Therefore, the observed deference in the prevalence of those studies could be attributed to the fact that some of them were facility-based and the participants were also at a plausibly higher chance of Mets development due to the comorbid psychiatric illness compared to those in the general community.

Additionally, the current study has included both adults and adolescents (> 17 years), unlike those previous studies which included only adults. The age spectrum difference between our study and the previous study may contribute to the discrepancies in the overall prevalence. At the study site, the prevalence of Mets is expected to be high in the urban area which has been demonstrated in other studies [25-27].

Previous studies revealed that the incidence of cardiovascular disease is high in older age; likewise, the prevalence of Mets showed an increasing trend with increasing age in this study. The finding is also consistent with reports from china and Brazil [28] [22]. The prevalence of Mets was found to increase among participants of advanced age, obesity, low physical activities, and unhealthy dietary practice. Such findings are warranting an early intervention to halt the progression of cardiovascular disease as Mets is a precursor of CVD in older age.

During a woman's lifetime, there are normal events such as puberty, pregnancy, and menopause, which are related to alterations in energy homeostasis and increased body fat due to hormonal effect Furthermore, conditions of severe metabolic stress and energy imbalance are commonly linked to alterations in Mets; In line with this, our observation showed a high prevalence of metabolic syndrome among female. This finding is similar to studies done on Africa women [27][29], Iranian women [30] and black African American women in the USA show a higher prevalence of Mets among women than men [25,31]. This finding could imply that Mets is a more prominent risk for developing cardiovascular disease among women than men and has been suggested by previous studies [32, 33]. In this regard, previous studies have also indicated the high relevance and burden of Mets among females and the need for preventive intervention to delay or prevent its occurrence [34, 35].

In our finding, the proportion of Mets was slightly higher among participants who also had high fasting blood glucose. Studies have reported that metabolic syndrome is a cluster of glucose intolerance, hypertension, dyslipidemia, and central obesity and it predicts diabetes independently of other factors [36] [30,37] [21]. People with metabolic syndrome are more likely to develop diabetes and this will increase the risk of developing cardiovascular disease.

A high proportion of the study participant was found to have low HDL protein which is consistent with a finding by another study done in Africa [29]. The most frequent abnormalities like low HDL-cholesterol, high LDL-cholesterol, and high total cholesterol are precursors of Mets and cardiovascular diseases.

Urban birth-place was significantly associated with Mets, which is in line with a systematic review and meta-analysis done in sub-Saharan Africa [27]. This is in contrast to other studies in similar settings which reported that relating it to the sedentary lifestyle (unhealthy dietary practice and lack of exercise are predominantly high in the urban area [38] as a plausible explanation to the observed high metabolic syndrome in an urban adult population. The study revealed alcohol consumption in the preceding 30 days has a significant association with Mets, a finding is in line with other studies [39] [28].

Frequent fasting was associated with high metabolic syndrome. Previous studies showed that intermittent fasting could protect against metabolic syndrome [40-42]. In most cases, prolonged fasting and avoidance of dairy product at the time of religious fasting is common in the study area.

The short-term improvements in some of the components of metabolic syndrome could be achieved by narrowing participants' eating period by time-restricted eating and vegan foods will reduce the chance of developing metabolic syndrome [40]. This study is strong in using large sample size, including both young and old participants, and in using WHO’s Step by step approach. For that reason, this study is adequately powered to show the magnitude of Mets across the spectrum of age. Since most of the previous studies of Mets were either hospital-based or targeting a specific patient population, this study has revealed a bigger picture of the problem by conducting the study in the general community. The limitation of this study is due to the cross-sectional study design; it will be difficult to make the temporal association between the factors found to be associated with metabolic syndrome in this study.

Conclusion and recommendation

The prevalence of metabolic syndrome is high in the urban community and relatively higher among females and increases with age. Though less prevalent than the general population, a significant proportion of adolescents and young adults have metabolic syndrome. A large proportion of participants have a low level of protective cholesterol, HDL. Low-density lipoprotein cholesterol, Body mass index, being female, urban birthplace, frequent alcoholic consumption for the last 30 days, and frequently fasting are factors associated with metabolic syndrome. We recommend both general and then targeted health education and health promotion interventions to be coordinated and employed by the relevant governmental and non-governmental stakeholders in order curb the emerging burden of non-communicable diseases in the resource-limited setting before it threatens the entire healthcare system.

Declarations

Consent for publication: Not Applicable

Availability of data and materials: We have to abide by the data sharing policy of the University of Gondar; nonetheless, we have included all important information regarding data presented (No additional data are available).

Competing interests: The authors declare no financial and non-financial competing interests

Authors’ contributions

 SMA, AT, NM designed the study;

 SMA, AT, NM performed the analysis and interpretation of data; and,

 SMA, AT, NM SA, and BA drafted and finalized the write-up of the paper.

 All authors prepared the draft manuscript, read and approved the final manuscript.

Acknowledgement

The authors are very grateful for the funding provided by the University of Gondar to conduct this study with full support, including training every study participant at each step of the study. We would also like to acknowledge the study participants for contributing to this research.

Authors' Information

We confirm that the manuscript has been read and approved by all named authors,

Figure 1. Distribution of IDF criteria based Metabolic Syndrome by age and sex among ≥18years old Gondar city residents, Northwest Ethiopia

Figure 2. Distribution of high total cholesterol level by age and sex among ≥18years old Gondar city

residents, Northwest Ethiopia

Figure 3: Distribution of high triglyceride level by age and sex among ≥18years old Gondar city residents, Northwest Ethiopia
