# Supplementary material for: Magnitude of metabolic syndrome in Gondar town, Northwest Ethiopia: A community-based cross-sectional study
Source: PLoS One. 2021 Oct 7;16(10):e0257306. doi: 10.1371/journal.pone.0257306 (PMC8496848; doi:10.1371/journal.pone.0257306)
Supplement: Attachment — Submitted filename: comments.docx [file pone.0257306.s004.docx]

Thanks for inviting me to review this manuscript entitled "Magnitude of Metabolic Syndrome in Gondar Town, northwest Ethiopia: A community-based cross-sectional study". This manuscript reported a community-based cross-sectional study in Ethiopia (n=3059), which provided a picture for the prevalence of MetS in East Africa. However, there are some areas needs improvements.

1. Careful editing is needed for the whole manuscript, such as the grammar, typos, e.g. Abstract-Conclusion and recommendation: line 6: “with high metabolic syndrome” ? The abbreviations should be explained with full names when they were firstly used, e.g. the IDF, NCD in the abstract.
2. Abstract: The study objectives were not presented clearly. “A community-based data on MetS” included what aspects? The results should be consistent with the objectives.
3. Background: The introduction should be presented more logistically.

-The rationales of conducting the current study should be clearly presented. From the current background, there were cross-sectional study, even population-based survey on MetS in Ethiopia. Why did the authors conducted the current one, using ATP and IDF definitions?

-P.10 of the submission file, 3^rd^ paragraph, the sentence of “Moreover, to fully comprehend the standing of Dyslipidemia in Ethiopia population-centered epidemiological documented information is necessary and essential.” is abrupt.

- Many points in the introduction were not clear, such as the “rapid growth”, “inequitable distribution” “climatic changes”, “cultural variables”.

4. Methods and Material:

-The time of conducting the study should be introduced.

-Sample size: why the author selected a “50% prevalence”? In the introduction, the prevalence ranged from 4.8%-20.3%.

-Sampling: how many people in each household were selected? “Finally, an individual from the

household was selected using a random lottery method. All the individuals in each household were given an identification number by the data collector.” The two sentences were confusing.

-Measurements: The measurement methods for WC, body weight and height should be introduced. The references for categorizing these variables should be provided individually.

- Data management and analysis: the references for using “a cut of point p-value < 0.20” should be provided.

5. Results:

- Why the authors used gender-and-age stratification in Table 1 and 2? This was not introduced previously?

- The whole data about MetS should be presented. For example, the mean, SD, and prevalence for BMI, WC, BP, FG, HDL, LDL, TG, and total cholesterol. The current manuscript only presented the categorical data (n, %). However, continuous data should be presented.

-Especially, as the IDF and ATP III used different WC criteria for abdominal obesity (94/80 or 102/88), prevalence of abdominal obesity following IDF and ATP III criteria should be provided. Otherwise, it is hard to understand the agreement of 91.7% between the two definitions.

-The risk factor for cardiovascular disease were not introduced previously. But Table 2 presented such data.

-The regression model included variables with p<0.20. Where were the p values from? t-tests or ANOVA, or Chi square tests? Please clarify.

-Multivariable analysis: One suggestion for the variable of marital status, the categories of separated, divorced, widowed could be combined.

- Table: there were typos in Table 1, e.g. Education status-Female: 214 (55,01); Marital status-Male : 29 (238).

- Figure: Figure 1 only presented the IDF criteria for age/gender prevalence of MetS, how about the ATP III criteria? Figure 2-4 presented the lipid files, how about other MetS components, like BP, WC, FG?

6. Discussion

- p.17 2^nd^ paragraph: It is not appropriate to make direct comparison “a considerable rise” about MetS prevalence, considering the study variances in study time, the populations, criteria.

-It is also confusing that “the current study has included both adults and adolescents (> 17 years)” p.17; “a significant proportion of adolescents and young adults have metabolic syndrome” p.20. The study criteria were “aged 18 or older”.

-The discussion on “ frequent fasting was associated with high metabolic syndrome” was not clear. The possible reasons of this finding were not discussed.

- The limitations in the current study were not discussed thoroughly.
