# Supplementary material for: Magnitude of metabolic syndrome in Gondar town, Northwest Ethiopia: A community-based cross-sectional study
Source: PLoS One. 2021 Oct 7;16(10):e0257306. doi: 10.1371/journal.pone.0257306 (PMC8496848; doi:10.1371/journal.pone.0257306)
Supplement: Attachment — Submitted filename: Point by point response of MetS 2 June 8_21.docx [file pone.0257306.s005.docx]

**To**: Plos one Editor-in-Chief

**Subject: -** **Point by point response for the comments and recommendations of our**

**reviewers**

**Title:**

**Reference**: MS:

Dear Editor-in-Chief

We are very grateful for the consideration of the manuscript. In accordance with the reviewers valuable comments and recommendations, we have revised the (MS 2) and we hereby re-submitting the revised work for your consideration.

| Question  No. | Question | Response |
| --- | --- | --- |
|  | **Essential revision** |  |
|  | **Editor** |  |
|  | English should be extensively revised.  -Introduction should be improved.  -Methodology needs important clarifications.  -Results and Discussion section should be more focused on the main results of the study. | Comments are well taken we have obtained further editorial assistance to improve the language from native English spiker  Moreover, revision have been made in the abstract, Introduction, Methods result and discussion section |
|  | 2. PLOS requires an ORCID iD for the corresponding author in Editorial Manager on papers submitted after December 6th, 2016. Please ensure that you have an ORCID iD and that it is validated in Editorial Manager. To do this, go to ‘Update my Information’ (in the upper left-hand corner of the main menu), and click on the Fetch/Validate link next to the ORCID field. This will take you to the ORCID site and allow you to create a new iD or authenticate a pre-existing iD in Editorial Manager. Please see the following video for instructions on linking an ORCID iD to your Editorial Manager account: https://www.youtube.com/watch?v=_xcclfuvtxQ | The ORCID iD of the corresponding author is updated and validated as per the directions.  Here is the updated ORCD Id: |
|  | 3. Please include additional information regarding the survey or questionnaire used in the study and ensure that you have provided sufficient details that others could replicate the analyses. For instance, if you developed a questionnaire as part of this study and it is not under a copyright more restrictive than CC-BY, please include a copy, in both the original language and English, as Supporting Information. | The questionnaire was directly adopted from the English version of the “WHO step by step approach for NCDs” and was translated to the local language called Amharic. We will also attach both the English and Amharic version if it helps as a supportive information. |
|  | 4. Thank you for stating the following in the Acknowledgments Section of your manuscript:  [The authors are very grateful for the funding provided by University of Gondarto conduct this study with full support,including training every study participant at each step of the study.]  We note that you have provided funding information that is not currently declared in your Funding Statement. However, funding information should not appear in the Acknowledgments section or other areas of your manuscript. We will only publish funding information present in the Funding Statement section of the online submission form.  Please remove any funding-related text from the manuscript and let us know how you would like to update your Funding Statement. Currently, your Funding Statement reads as follows:   [No The funders had no role in study design, data collection and analysis, decision to publish, or preparation of the manuscript] | The authors accept the comments and have removed the funding related information from the acknowledgment part.  University of Gondar, a public higher education institute in Ethiopia, encourages the research culture of it’s academicians by arranging competitive partial grants for research projects. Less than 10% of research proposals get the partial grant (which does not include publication fee) and our research was granted through such small local grant.  Therefore, the funding statement can be kept as it is as we have now omitted the funding information from the acknowledgement part.  i.e.   [No The funders had no role in study design, data collection and analysis, decision to publish, or preparation of the manuscript] |
|  | **Reviewer 1** |  |
|  | Reviewer #1: Thanks to the authors It is better to correct and improve the English writing of the text.In the methodology section, the method of measuring the sample size is not completely clear Please see the word's attached file. | Comments are well noted, revision and correction are made.  we have obtained further editorial assistance to improve the language |
|  | **Reviewer #2:** Thanks for inviting me to review this manuscript entitled "Magnitude of Metabolic Syndrome in Gondar Town, northwest Ethiopia: A community-based cross-sectional study". This manuscript reported a community-based cross-sectional study in Ethiopia (n=3059), which provided a picture for the prevalence of MetS in East Africa. Please see the attachment froe detailed comments. | comments incorporation and revision done as per the comments. Please see the details below: |
|  | **Reviewer #3** |  |
|  | 1. The introduction section does not amply describe a need to conduct a prevalence study on MetS on the said population. The authors themselves hinted on various such studies done already in this population and as per them, those seemed to have gaps in terms of lifestyle and anthropological perspectives; however, the authors in this study lacked to mention details on how those gaps were addressed in this study. | Comments are well taken,  Additional point and revision is done in the background section (please see the track change in the background) |
|  | 2. The other concern regarding this study is the methodology used as the sampling design does not adequately cover the population due to the random lottery method used. The survey can also fail to cover the population as it does not categorize the people living in both standard and non-standard dwellings. This is an important factor for any community- based study. | The study participants were resident of Gondar City, assuming a similar characteristics (homogenies) in terms of dietary practiced, and lifestyle;  For the sample size determination we used a separate sample size calculation/ determination considering the age and sex category, we also follow simple randomly sampling in order to select the representative study participant from each sub-city for age and sex group |
|  | 3. Other issue about the methodology is the ATPIII criteria which is best predictor of the cardiovascular events but in elderly people. The conclusion drawn from the study thus cannot be extrapolated to other age-groups. Besides, the sample size calculation shown in the methodology section to select a representative sample for prevalence studies (especially when prevalence’s of >30% MetS have been reported in some studies) for a population of about 109 million needs revisiting. | It is a well known that age by itself is an established predictor (non-modifiable risk factor) of cardiovascular event. ATPIII is one of the criteria used to diagnosed metabolic syndrome (various combinations of components of modifiable risk factors). We don’t is not a predictor by itself.  Having said that, we have estimation for each age category please see the newly add figure 5, generalization only for the respective category  Yes, conclusion drawn from the study cannot be extrapolated to other age-groups, the whole essence of this study is to show the distribution of MetS at different age group using the two criteria (ATPIII & IDF); the high prevalence is observed in older age group and we have seen association between the observed high prevalence with cardiovascular disease. |
|  | 4. The measurement section is a little confusing to the readers as the first sentence first sentence itself states that WHO and IDA criterion were used to classify hypertension while as per the abstract and rest of the manuscript, NCEP ATPIII or IDF were used to classify all the components of MetS. Also, the way of presenting the questionnaire details in the last paragraph of measurement section needs revisiting and should be presented in a more scientific manner. | Correction done on WHO and IDF to avoid confusion  We use WHO step wise approach to collect data while letter we use ATP III and IDF criteria to classify MetS status (please see the revision in abstract and measurement section) |
|  | 5. The result section should have been presented as and according to the data given in the tables and highlighting on the main findings which needed to be discussed in the discussion section. Also, the results should have been presented in accordance with the study objective which was to derive at the prevalence of MetS in the said population. MetS, according to the criteria used, has components like “central obesity”, “hypertension”, “hypertriglyceridemia”, “low HDL-cholesterol” and “hyperglycemia” and thus focus should have been on these components and the full MetS rather than describing hypercholesteromia, high LDL-C, etc. | Comments are well taken and revision done focusing on the key findings, and its order  (please see the rearrangement in the revised version with a track change) |
|  | 6. The discussion lacks the details of the defense of the observations and findings in the current study. For example, the initial statements in the discussion relates the findings in the current study in line with a study (reference # 20) while the sample size in that study was 314 and the prevalence of MetS observed was 59.9% and 70.1% according to IDF and NCEP criteria’s respectively. Besides, the authors struggled in explaining the reason for such a low prevalence of MetS (as we have numerous reports of much higher prevalence of MetS in the general population worldwide). | Considering your valuable comment we have tried to incorporate findings from Ethiopia to show context relation or difference with possible explanation and its implication (please see the change in the second paragraph of the discussion section) |
|  | 7. Lastly, there are numerous grammatical mistakes in the entire manuscript which should be looked at by the authors seriously before resubmission. Besides, the statistics done should be revisited e.g. in table 3, O.R. for high LDL-C is mentioned as 1.01 with 95% C.I. of 1.00 to 1.01 and associated p-value <0.001 which is not possible and needs relooking. Also, none of the three tables given in this manuscript specifically deal with data by which MetS was calculated apart from calculating the five components of MetS as discussed before. | Comments are well taken, correction done in addition figure 5 is incorporated to address the issue (Please see figures 5) |
|  | Reviewer 2 |  |
|  | 1. Careful editing is needed for the whole manuscript, such as the grammar, typos, e.g. Abstract-Conclusion and recommendation: line 6: “with high metabolic syndrome” ? The abbreviations should be explained with full names when they were firstly used, e.g. the IDF, NCD in the abstract. | **Comments are well taken, correction done in the revised section across the MS,**  The abbreviations are explained in full names in the abstract |
|  | 2. Abstract: The study objectives were not presented clearly. “A community-based data on MetS” included what aspects? The results should be consistent with the objectives. | Done according to the suggestions. (comments are included in a track change) |
|  | 3. Background: The introduction should be presented more logistically.   -The rationales of conducting the current study should be clearly presented. From the current background, there were cross-sectional study, even population-based survey on MetS in Ethiopia. Why did the authors conducted the current one, using ATP and IDF definitions? | Comments are well taken additional points are incorporated to show the research gap (what is unknown in the background section) |
|  | -P.10 of the submission file, 3^rd^ paragraph, the sentence of “Moreover, to fully comprehend the standing of Dyslipidemia in Ethiopia population-centered epidemiological documented information is necessary and essential.” is abrupt. | Correction done as suggested |
|  | - Many points in the introduction were not clear, such as the “rapid growth”, “inequitable distribution” “climatic changes”, “cultural variables”. | Comments are well taken those points are deleted for better clarity and focus |
|  | 4. Methods and Material:   -The time of conducting the study should be introduced. | Well noted It’s 2018 |
|  | -Sample size: why the author selected a “50% prevalence”? In the introduction, the prevalence ranged from 4.8%-20.3%.  -Sampling: how many people in each household were selected? “Finally, an individual from the  household was selected using a random lottery method. All the individuals in each household were given an identification number by the data collector.” The two sentences were confusing. | The referred estimation sample size was taken in a pooled estimation prevalence, but ours sample size determination was done for each category of age and sex separately, and reported separately for those categories  Based on the suggestion revision done in the sapling process |
|  | Measurements: The measurement methods for WC, body weight and height should be introduced. The references for categorizing these variables should be provided individually. | Comments are incorporated in the revised MS measurement section |
|  | - Data management and analysis: the references for using “a cut of point p-value < 0.20” should be provided. | **For the multivariable analysis to include the marginal confounder we use a wider cut of point ≤ 0.20 (rule of thumb)** |
|  | 5. Results:   - Why the authors used gender-and-age stratification in Table 1 and 2? This was not introduced previously? | Study clearly indicate age and sex are predictors of MetS, in this study we want to show the distribution of MetS (prevalence of MetS across different age and sex group to have better information about the burden of the problem for public health intervention  Some ideas are also introduced in the background section |
|  | - The whole data about MetS should be presented. For example, the mean, SD, and prevalence for BMI, WC, BP, FG, HDL, LDL, TG, and total cholesterol. The current manuscript only presented the categorical data (n, %). However, continuous data should be presented. | Comments are well taken additional table 4 is provided to address the comment |
|  | -Especially, as the IDF and ATP III used different WC criteria for abdominal obesity (94/80 or 102/88), prevalence of abdominal obesity following IDF and ATP III criteria should be provided. Otherwise, it is hard to understand the agreement of 91.7% between the two definitions. | Prevalence of abdominal obesity following IDF and ATP III criteria, the proportion of Waist circumference using ATP III criteria was 12.7% [95%CI: 11.5, 13.9], and that of IDF was 26.9% [95%CI: 25.3, 28.5].  The agreement analysis/ computed was for MetS, but WC is one of the composite variable used to measure MetS; |
|  | -The risk factor for cardiovascular disease were not introduced previously. But Table  2 presented such data.  -The regression model included variables with p<0.20. Where were the p values from? t-tests or ANOVA, or Chi square tests? Please clarify. | The focus is not to discuss the risk factors for cardiovascular but just to show the whole picture of the burden  Now we moved the table in to additional date if its not ok we can introduce detail in the main MS.  The regression model we included variables with p<0.20 following univariate analysis in a Chi square tests. |
|  | -Multivariable analysis: One suggestion for the variable of marital status, the categories of separated, divorced, widowed could be combined. | Comments are well taken we recode to combine, the categories of separated, divorced, and widowed marital status and re run the regression (see table 3) |
|  | - Table: there were typos in Table 1, e.g. Education status-Female: 214 (55,01); Marital status-Male : 29 (238). | Comments are well taken and correction done as suggested |
|  | - Figure: Figure 1 only presented the IDF criteria for age/gender prevalence of MetS, how about the ATP III criteria? Figure 2-4 presented the lipid files, how about other MetS components, like BP, WC, FG? | We have incorporated additional figure (figure 5) to address the point  About other about other MetS components, like BP, WC, FG we have concern about the scope/focus we may end up with a lot of figure, we also show the mean in the new table 2 |
|  | 6. Discussion   - p.17 2^nd^ paragraph: It is not appropriate to make direct comparison “a considerable rise” about MetS prevalence, considering the study variances in study time, the populations, criteria. | Revision is done based on the feedback |
|  | -It is also confusing that “the current study has included both adults and adolescents (> 17 years)” p.17; “a significant proportion of adolescents and young adults have metabolic syndrome” p.20. The study criteria were “aged 18 or older”. | The current study is included only age 18 and above we do not have study participant from adolescents age 17 or less  Correction is done for the word adolescents |
|  | -The discussion on “ frequent fasting was associated with high metabolic syndrome” was not clear. The possible reasons of this finding were not discussed. | Comments are well taken additional clarity point is included to address the comment |
|  | - The limitations in the current study were not discussed thoroughly. | Done as commented (please the point in the limitation section |

With Best Regard!!

The Authors
