# Supplementary material for: Magnitude of metabolic syndrome in Gondar town, Northwest Ethiopia: A community-based cross-sectional study
Source: PLoS One. 2021 Oct 7;16(10):e0257306. doi: 10.1371/journal.pone.0257306 (PMC8496848; doi:10.1371/journal.pone.0257306)
Supplement: Attachment — Submitted filename: revised version 2 comments-Qun.docx [file pone.0257306.s006.docx]

Thanks for inviting me to review the revised manuscript. The authors made some revisions following the previous comments. However, there are still some questions needs clarification and improvements. The authors should point out the detailed location of each revision in the responses (e.g. page no. Paragraph, line).

1. Careful editing is still needed for the whole manuscript. Please pay attention to the following:

--It should be “metabolic syndrome” but not “Metabolic syndrome” if this word is not in the beginning of the sentence.

-P.41 the last 2^nd^ line, it should be “such as obesity...” .

- Measurement: “Finally, biochemical tests (fasting blood glucose levels....) were carried out. Impaired fasting blood glucose levels (IFG) was identified if the fasting blood glucose levels were between 110 and 125 mg/dl.

- Results Session: “A unit increase in LDLC/ BMI...will increase the prevalence of metabolic Syndrome by XX%. ”

-Table 2: “Standard deviation”

- Discussion: P.50, line 2 : “ a higher prevalence”

2. Sample size: the rationales or references of using a “50% prevalence” for the age-stratified sample size calculation should be provided.
