# Supplementary material for: Magnitude of metabolic syndrome in Gondar town, Northwest Ethiopia: A community-based cross-sectional study
Source: PLoS One. 2021 Oct 7;16(10):e0257306. doi: 10.1371/journal.pone.0257306 (PMC8496848; doi:10.1371/journal.pone.0257306)
Supplement: Attachment — Submitted filename: Ctd_Point by point response MetS 27-8-21.doc [file pone.0257306.s007.doc]

**To**: Plos one Editor-in-Chief

**Subject: -Point by point response for the comments and recommendations of**

**reviewers**

**Title:**

**Reference**: MS:

Dear Editor-in-Chief

We are very grateful for the consideration of the manuscript. In accordance with the reviewers valuable comments and recommendations, we have revised the (MS 4) and we hereby re-submitting the revised work for your consideration.

PONE-D-21-02096R1
Magnitude of Metabolic Syndrome in Gondar Town, northwest Ethiopia: A community-based cross-sectional study
PLOS ONE

| Question  No. | Question | Response |
| --- | --- | --- |
|  | **Essential revision** |  |
|  | **Editor** |  |
|  | Thanks for inviting me to review the revised manuscript. The authors made some revisions following the previous comments. However, there are still some questions needs clarification and improvements. The authors should point out the detailed location of each revision in the responses (e.g. page no. Paragraph, line).   1. Careful editing is still needed for the whole manuscript. Please pay attention to the following: | The authors accept the comments and have obtained further edition to improve the language. |
|  | --It should be “metabolic syndrome” but not “Metabolic syndrome” if this word is not in the beginning of the sentence. | comments well taken revision done as per the comments |
|  |  | comments well taken revision done as per the comments, for farther clarity in the revised version, |
|  | -P.41 the last 2^nd^ line, it should be “such as obesity...” . | Thank you for the comment, correction done (please see the track change in the revised version) |
|  | - Measurement: “Finally, biochemical tests (fasting blood glucose levels....) were carried out. Impaired fasting blood glucose levels (IFG) was identified if the fasting blood glucose levels were between 110 and 125 mg/dl. | Thank you for the comment, correction done (please see the track change in the revised version) |
|  | - Results Session: “A unit increase in LDLC/ BMI...will increase the prevalence of metabolic Syndrome by XX%. ” | Comments are well taken; correction done |
|  | - Table 2: “Standard deviation” | Thank you for the comment, spelling correction done |
|  | - Discussion: P.50, line 2 : “ a higher prevalence” | Thank you for the comment, spelling correction done |
|  | Sample size: the rationales or references of using a “50% prevalence” for the age-stratified sample size calculation should be provided | Comments are well taken, the reference is cited : World Health Organization. Noncommunicable Diseases and Mental Health Cluster. (‎2005)‎. WHO STEPS surveillance manual : the WHO STEPwise approach to chronic disease risk factor surveillance / Noncommunicable Diseases and Mental Health, World Health Organization. World Health Organization. <https://apps.who.int/iris/handle/10665/43376>, ISBN: 9241593830: |

With Best Regard!!

The Authors
